# Pandemic (H1N1) 2009 influenza: Experience from a critical care unit in India

**DOI:** 10.4103/0972-5229.74177

**Published:** 2010

**Authors:** Jyoti N. Sahoo, Banani Poddar, Afzal Azim, Ratender K. Singh, Mohan Gurjar, Arvind K. Baronia

**Affiliations:** **From:** Department of Critical Care Medicine, Sanjay Gandhi Postgraduate Institute of Medical Sciences, Lucknow, Uttar Pradesh, India

**Keywords:** Acute respiratory distress syndrome, human, H1N1 subtype, influenza A virus

## Abstract

This case series details our experience with seven patients with pandemic (H1N1) 2009 influenza from an intensive care unit in India.All the patients had respiratory failure requiring ventilation except one;two patients developed pneumothorax. Of the seven patients, two died (28.5%) and five recovered. Four patients had co-morbid conditions and one was morbidly obese; all the five patients were discharged alive.

## Introduction

On May 16, 2009, the first case of pandemic (H1N1) 2009 influenza infection in India was reported in a traveler from United States in Hyderabad; by October 2009, H1N1 infection was reported from all parts of India. The first casualty from pandemic (H1N1) 2009 influenza infection in India was reported to be on August 3, 2009 from Pune.[1]

Current prediction estimates that during the pandemic wave, 12–30% of the population will develop clinical flu (compared with 5–15% for seasonal flu), with 4% requiring hospital admissions and one in five requiring critical care.[2,3] These latter patients rapidly develop severe progressive respiratory failure, which is sometimes associated with failure of other organs and marked worsening of underlying airway disease.[4]

While several studies from the West describing the severely ill ICU patients with pandemic (H1N1) 2009 disease are available, no such studies are available from India. During the peak period between November–December 2009 and January 2010, we admitted seven patients with pandemic (H1N1) 2009 influenza viral infection with severe hypoxemia. We present the clinical characteristics of these patients.

## Methods

Data from seven consecutive patients with pandemic (H1N1) 2009 viral disease with severe hypoxemia, admitted to the intensive care unit of a tertiary care hospital in India, were included. We retrospectively reviewed medical charts, radiology and laboratory findings. All the study patients had influenza-like illness with opacities found on chest radiography and were presumed or laboratory-confirmed H1N1 viral infection cases.

## Results

The demographic characteristics of the patients are shown in Table 1. As can be seen in the table, five patients were below the age of 40 years and six were females; none of the females was pregnant or recently gravid. Four of the seven patients had co-morbidities [hypothyroidism 2, chronic obstructive pulmonary disease (COPD) 1, coronary artery disease 1, congenital heart disease 1] and one was morbidly obese; however, none of these patients died. All had a history of respiratory symptoms such as cough and sore throat, in addition to fever. They were admitted after a median duration of 7 days (4–18 days) from the onset of symptoms. Four patients were transferred from another ICU to our center.

**Table 1:** Demographic profile

Age (years)	
Median	35
Range	24–65
All patients: no./total no. (%)	
21–40 years	55/7 (71.4)
41–60 years	1/7 (14.28)
≥61 years	1/7 (14.28)
Female sex: no./total no. (%)	6/7 (85.7)
Patients who died: no./total no.	
21–40 years	2/5
41–60 years	0/1
≥61 years	0/1
Presence of comorbid condition: no./total no. (%)	4/7 (57.14%)
Days from onset of symptoms to hospital admission:median (range)	7 (4–18)
Days from admission to death: median (range)	13 (10–16)
Transferred from other ICU: no./total no.	4/7

The admission profile of the patients is shown in Table 2. All presented with breathing difficulty, and hypoxemia was found with initial PaO_2_/FiO_2_ratio 40–200 (median 60). All the patients had bilateral patchy alveolar opacities radiologically, predominantly in the lower lobes; in four patients, three or four lung quadrants were affected. The median lung injury score at admission was 2.5 (0.5–3.5). The initial PaCO_2_ was 25–45 mm Hg in five patients, in one patient it was 52 mm Hg, and in a COPD patient it was 84 mm Hg. The illness severity as characterized by Acute Physiology and Chronic Health Evaluation (APACHE II) score was mild to moderate with a median score of 9 (range 7–14). Some patients had involvement of other organs at admission as evidenced by Sequential Organ Failure Assessment (SOFA) score of 1–8 (median 5). Of the seven patients, two had leukopenia and five had leukocytosis (>11,000 cells/mm^3^). Four patients had lymphopenia (<1000 lymphocytes/mm^3^). All had elevated procalcitonin level ranging from 1.09 to 6.91 ng/ml. Five patients had high serum lactate level (>4.5 mmol/l).

**Table 2:** Admission characteristics; laboratory data and severity of illness

Parameters	Median (range)
APACHE-II score	9 (7–14)
SOFA score	5 (1–8)
Initial LIS	2.5 (0.5–3.5)
Initial PaO_2_/FiO_2_ ratio	60 (40–200)
Initial PaCO_2_	40 (25–84)
Lactate (mg/dl)	22.49 (7.71–61)
Total leukocyte count(cells/*μ*l)	16.2 (2.7–35)
Leukocytosis (>11,000/μl): no./total no.	5/7 (71.4%)
Leukopenia (<4000/μl): no./total no.	2/7 (28.57%)
Lymphopenia (<1000 lymphocytes/mm3)	4/7 (57.14%)
Procalcitonin (ng/ml)	4.39 (1.09–6.90)
Bacterial colonization at admission: no./total	4/7 (57.14%)

APACHE II=Acute physiology and chronic health evaluation; SOFA=Sequential organ failure assessment score; LIS=Lungs injury score; PaO_2_/FiO_2_=Arterial partial pressure of oxygen concentration fraction inspired oxygen concentration

Polymerase chain reaction (PCR) for H1N1 virus was confirmatory in six patients. In one patient, the clinical course was very suggestive of this disease and hence she has been included in this case series. The patient came to our ICU several days after the onset of her illness and testing in our hospital was negative. Tracheal aspirates were cultured at admission, and as clinically indicated, four patients were found to have secondary bacterial infection at admission [Table 2]. All were treated with Oseltamivir 75 mg twice daily for 10 days along with antibiotics as decided by the ICU physician.

The supportive therapies given in the ICU are detailed in Table 3. Respiratory failure progressed rapidly in all, and six out of seven patients required mechanical ventilation within a median of 4 hours after admission (range 0–10 in five patients, 120 hours in one patient). Three patients were on noninvasive ventilation [bilevel positive airway pressure (BiPAP)], of which two were later put on invasive ventilation. One patient had received non-invasive positive pressure ventilation (NIPPV) for 5 days before being transferred to our ICU. All six patients requiring mechanical ventilation were ventilated using lung protective ventilation strategies [targeting tidal volume 4–6 ml/kg ideal body weight (IBW) and plateau pressure less than 30 cm of H_2_O]; three required positive end expiratory pressure of 14–16 cm of H_2_O. In these six patients with severe hypoxemia, neuromuscular paralysis was used in five, and prone position ventilation in three patients. Duration of mechanical ventilation ranged from 3 to 16 days (median 12 days). Two of these developed pneumothorax while on mechanical ventilation, requiring intercostal tube drainage; in one, the peak inspiratory pressure was 40 cm H_2_O prior to pneumothorax, whereas in the other, the peak pressure was 35 cm H_2_O. The former patient died, while the latter survived. Corticosteroids were administered to four patients, one with COPD and three with severe acute respiratory distress syndrome (ARDS); of these four, two died.

**Table 3:** ICU management and outcome

No. received BiPAP	3
Mortality: no./total no.	1/3
No. of patients on mechanical ventilation: no./total no.	6/7 (85.7%)
Hours from hospital admission to intubation and mechanical ventilation: median (range)	9 (1–120)
Days on mechanical ventilation: median (range)	12 (3–16)
Maximum PEEP(cm of H_2_O)	
10–13: no./total no.	3/6
14–16: no./total no.	3/6 (50%)
Prone ventilation (MV patients): no./total no.	3/6 (50%)
Neuromuscular paralysis use: no/total no.	5/6 (83.3%)
Pneumothorax: no./total no.	2/7 (28.57%)
Use of corticosteroids: no./total no.	4/7
Use of vasopressors: no./total no.	5/7
At admission	1/5
During ICU stay	4/5
MODS: no/total no.	5/7 (71.4%)
Mortality	2/5 (40%)
Days in ICU: median (range)	16 (4–28)
Mortality overall: no./total no.	2/7 (28.57%)
On MV	2/6 (33.3%)

BiPAP=Bilevel positive airway pressure; PEEP=Positive end expiratory pressure

Fluid non-responsive shock developed during the course of ICU stay in five of seven patients requiring norepinephrine infusion. Three patients developed acute kidney injury; one died and none required renal replacement therapy. Five patients had multiorgan system failure, of whom two died. Bacterial colonization was found in four patients: *Acinetobacter baumanii* in all the four patients, *Pseudomonas aeruginosa* in two and *Klebsiella pneumoniae* in one.

Of the seven patients, two died (28.5%) and five recovered [Table 4]. Of the six patients on mechanical ventilation, 2 died (33.3%). Both the patients died due to refractory hypoxemia. Duration of intensive care stay ranged from 4 to 28 days (median 16 days).

**Table 4:** Comparison between survivors and non survivors

	Survivors (median)	Non survivors (median)
Age (years)	38	29.5
Sex (M:F)	1:4	0:2
Onset of symptoms to hospital admission (days)	7	15
APACHE-II	9	10.5
LIS	1.5	3
Hospital admission to intubation (hours)	10	≤5
Worst PaO_2_/FiO_2_	130	60
PaCO_2_ (mm of Hg)	36	45
Lactate (mg/dl)	19.4	47.5
Procalcitonin (ng/ml)	1.13	6.29
Days of mechanical ventilation MV	10	13
Maximum PEEP (mm of Hg)	12	15
AKI (no, %)	2, 40	1, 50

APACHE II=Acute physiology and chronic health evaluation; LIS=Lung injury score; PaO2/FiO2=Arterial partial pressure of oxygen/fraction of inspired oxygen concentration; PEEP=Positive end expiratory pressure; AKI=Acute kidney injury

## Discussion

In late March and early April 2009, an outbreak of H1N1 influenza A virus infection was detected in Mexico with subsequent cases observed in many other countries.[5] On June 11, 2009, World Health Organization (WHO) raised its pandemic level to the highest level, phase 6, indicating widespread community transmission in at least two continents. By October 2009, 191 countries and territories reported more than 375,000 laboratory confirmed H1N1 infected cases with more than 4500 deaths.[3]

The patients with H1N1 viral disease admitted to our ICU were below the age of 40 years, except two. Similarly, Western studies have also shown that critical illness resulting from pandemic 2009 (H1N1) occurs in young adults.[4] Six patients (85.7%) were females. Kumar *et al*. also found that a higher proportion of women required intensive care.[6]

Various studies have shown that patients requiring hospitalization for 2009 H1N1 infection are those with underlying medical conditions like asthma, chronic obstructive airway disease, morbid obesity [body mass index (BMI) > 30 kg/m^2^], chronic heart conditions and immunosupression.[7] Out of seven patients, five had co-morbid conditions, all of whom survived.

Patients were admitted at a median of 7 days after the onset of symptoms and were intubated soon after hospital admission. This is in keeping with other studies,[4] which showed that in severe cases, patients generally began to deteriorate around 4–6 days after the symptom onset; however, respiratory failure progressed rapidly requiring mechanical ventilation.[4] Hypoxemia and chest radiography consistent with ARDS is the hallmark of patients requiring intensive care.[7] Early invasive ventilation is recommended, and using noninvasive ventilation as an interim measure may worsen the outcome.[8]

In view of the severe ARDS, corticosteroids have been used in ICU patients with pandemic H1N1.[9] However, whether this is beneficial or not has not been resolved as prolonged use of high-dose corticosteroids in these patients may also increase the susceptibility to opportunistic infections.[4] Corticosteroids may also increase the viral shedding time.[9]

In addition to direct viral pneumonia, pneumonia caused by coinfection with bacteria can also contribute to a severe rapidly progressive illness.[4] Bacteria frequently reported include *Streptococcus* and *Staphylococcus*; these bacterial coinfections are more frequent than initially thought.[4] In our case series, *Acinetobacter* was the commonest organism found, probably because Gram-negative organisms are the commonest nosocomial pathogens in Indian ICUs. Four of our patients were colonized right at admission due to exposure to another health care set-up.

The need for vasopressor therapy was often associated with the requirement for high sedation and neuromuscular paralysis to assist ventilation.[2] In our case series also, we found five patients requiring vasopressor support. Five patients developed multi organ dysfunction syndrome (MODS) during ICU stay. The commonest organ system involved was renal.

The ICU mortality in various studies varied from 10 to 38% and was found to be higher in those requiring invasive ventilation up to 58%.[4–7] The adverse prognostic factors include older age, BMI > 30, need for mechanical ventilation at admission and presence of acute kidney injury or coinfection.[10] Two of our patients died (28.5%) from refractory hypoxemia, both of whom required mechanical ventilation at admission. None of our patients received extracorporeal membrane oxygenation (ECMO) support; which has been shown to improve the outcome.[11]

## Conclusions

This series illustrates the presentation and management difficulties of pandemic H1N1 influenza disease in the intensive care unit. The intensivist must be prepared for highly individualized ventilator management, depending on severity of hypoxemia, and hemodynamic instability. Significant sedation and even neuromuscular blockade may be necessary to manage severe hypoxemia. Refractory hypoxemia is the commonest cause of mortality.

Note: At the time of submission of this manuscript, there were no Indian studies. Subsequently, a study has been published in the Apr-Jun issue of IJCCM, the ref for which is Chacko J, Gagan B, Ashok E, Radha M, Hemanth HV. Critically ill patients with 2009 H1N1 infection in an Indian ICU. IJCCM 2010; 14(2): 77-82
